# Nutritionally acquired immunodeficiency must be addressed with the same urgency as HIV to end tuberculosis

**DOI:** 10.1186/s44263-023-00035-0

**Published:** 2024-01-12

**Authors:** Madolyn R. Dauphinais, Kobto G. Koura, Prakash Babu Narasimhan, Saurabh Mehta, Julia L. Finkelstein, Scott K. Heysell, Pranay Sinha

**Affiliations:** 1 Boston Medical Center, 801 Massachusetts Ave, Crosstown Center, Suite 2021A, Boston, MA 02118 USA; 2 International Union Against Tuberculosis and Lung Disease, Paris, France; 3 Communauté d’universités et établissements Sorbonne Paris Cité, Faculté des Sciences Pharmaceutiques et Biologiques, Université Paris Descartes, Paris, France; 4 École Nationale de Formation des Techniciens Supérieurs en Santé Publique et en Surveillance Epidémiologique, Université de Parakou, Parakou, Benin; 5 Department of Clinical Immunology, Jawaharlal Institute of Postgraduate Medical Education and Research, Puducherry, India; 6 Center for Precision Nutrition and Health, Cornell University, Ithaca, NY USA; 7 St John’s Research Institute, Bengaluru, Karnataka India; 8 Division of Nutritional Sciences, Cornell University, Ithaca, NY USA; 9 Division of Epidemiology, Department of Population Health Sciences, Weill Cornell Medicine, New York, NY USA; 10 Division of Infectious Diseases and International Health, University of Virginia, Charlottesville, VA USA; 11 Section of Infectious Diseases, Boston University Chobanian & Avedisian School of Medicine, Boston, MA USA

**Keywords:** Tuberculosis, HIV, Malnutrition

## Abstract

Tuberculosis (TB) is the leading infectious killer worldwide, with 10.6 million cases and 1.6 million deaths in 2021 alone. One in 5 incident TB cases were attributable to malnutrition, more than double the fraction attributed to HIV. Like HIV, malnutrition is a cause of secondary immunodeficiency and has even been dubbed nutritionally acquired immunodeficiency syndrome (N-AIDS). However, malnutrition remains the neglected cousin of HIV in global TB elimination efforts. Malnutrition increases the risk for TB progression, increases disease severity, and worsens TB treatment outcomes. Thus, it is both a TB determinant and comorbidity. In this perspective, we discuss decades of data to make the case that N-AIDS, just like HIV/AIDS, also deserves special consideration in the TB elimination discourse. Fortunately, malnutrition is a modifiable risk factor and there is now empirical evidence that addressing nutrition can help us curb the TB pandemic. Recognizing malnutrition as a key determinant and comorbidity is key to detecting and treating the missing millions while also preventing additional millions from suffering TB disease.

## Background

What would have happened if we never discovered the human immunodeficiency virus (HIV)? We would be mystified as to why certain groups died disproportionately of devastating opportunistic infections—tuberculosis (TB) chief among them. With higher rates of progression, a wider range of clinical manifestations, and worse clinical outcomes, inaction on HIV would have doomed global efforts to reduce TB incidence and mortality [1]. A plethora of research spurred by people affected by HIV and the activist community established HIV as both a determinant and comorbidity of TB. The recognition of the HIV/TB syndemic led to prompt integration of HIV screening and care into standard TB management protocols [2]. HIV and TB are now firmly linked in the minds of clinicians, researchers, and policymakers. However, HIV is not the only acquired immunodeficiency syndrome driving the TB epidemic.

The leading cause of secondary immunodeficiency worldwide is malnutrition [3]. Malnutrition-related immunosuppression has even been dubbed as nutritionally acquired immunodeficiency syndrome (N-AIDS) [4]. With blunted adaptive and innate immunity, malnourished individuals, like persons living with HIV, are at increased risk of infections [3]. Further, infectious diseases can also present more aggressively and atypically in malnourished persons. The link between poor nutrition and TB has been recognized for at least a century [5].

Indeed, in the pre-antibiotic era, proper nutritional support was the cornerstone of TB therapy in sanatoria [6]. Natural experiments during the two world wars linked malnutrition and TB [7]. The malnourished were identified as a key and vulnerable population in the fight against TB. The Papworth study in England demonstrated the role of addressing social determinants like housing and nutrition in reducing TB progression among household contacts of persons with TB [8]. In Chile, addressing malnutrition was a key component of a national TB elimination program [9]. The USA, England, and Wales all saw dramatic reductions in TB in the first half of the 20th century before any medications or vaccines were available for TB [10]. Thomas McKeown and others have attributed these reductions to socioeconomic progress—better living conditions and better nutrition [11].

Unfortunately, nutrition fell out of focus with the introduction of successful combination drug therapy for TB in the 1960s [6]. TB elimination efforts became progressively siloed in the biomedical realm [12]. However, as the impact of antibiotics on mortality reduction from TB increased, the impact of malnutrition on TB disease progression never really receded. Today, it is estimated that 19% of TB incidence is attributable to malnutrition (population attributable fraction [PAF]: 19%), more than double that of HIV at 7.6% [2]. Some of the lessons learned from HIV/AIDS can be applied to address the impact of N-AIDS on TB.

In this perspective, we make the case that recognizing malnutrition as a serious determinant and comorbidity of TB will help detect, treat, and prevent TB and expedite the end of the TB pandemic.

### Detection of TB

Finding the missing millions is a key step in eliminating TB. In 2020, of the nearly 10 million estimated cases of tuberculosis disease, only 5.8 million cases were diagnosed and reported.^2,12^ Data also show that individuals have active and transmissible TB for 4 to 14 months before diagnosis, which highlights an opportunity to interrupt the individual’s disease course and curb population-wide spread earlier [2]. Weight loss is a cardinal, but insidious symptom of TB [13]. Persons with TB (PWTB) often present with weight loss prior to the onset of more prominent symptoms. In high TB-burden countries such as India, cohort studies have reported that more than 50% of PWTB were malnourished at the time of diagnosis [14, 15]. The World Health Organization (WHO) currently recommends systematic screening for TB in populations where the prevalence of TB exceeds 0.5% [16]. There is ample evidence to suggest that TB may be more prevalent among malnourished individuals. In Myanmar, for instance, the prevalence of TB in persons with body mass index (BMI) <18.5 kg/m^2^ was 1.2% [17]. Similarly, India’s National TB Prevalence Survey from 2021 found that individuals with a BMI <18.5 kg/m^2^ had a higher likelihood of TB positivity [18]. In Tamil Nadu, India, the prevalence of bacteriologic positive TB among individuals with BMI <18.5kg/m^2^ was more than fourfold higher compared to individuals with BMI ≥18.5kg/m^2^ or 892 vs. 198 cases per 100,000 population [19]. The prevalence of TB among malnourished individuals meaningfully exceeds the WHO’s threshold for systematic screening. Indeed, malnourished individuals had 4.48 times greater odds (95% confidence interval [CI] 3.26–6.15), for TB detection, after adjusting for age, sex, alcohol use, tobacco use, cooking fuel exposure, history of TB diabetes, and HIV. Malnourished individuals also had a fourfold higher rate of smear-positive TB as compared to well-nourished individuals (365/100,000 vs. 86/100,000). This speaks to the importance of systematic screening for TB among malnourished individuals to prevent further transmission.

Importantly, the abovementioned studies only speak to the prevalence of clinical TB among malnourished persons. There is reason to believe that subclinical TB disease also drives transmission within communities [20]. The prevalence of subclinical disease remains ill-defined in malnourished populations and must be explored through screening studies that test for TB disease using chest radiographs, sputum culture, and nucleic acid amplification testing regardless of clinical symptoms. Additionally, we must consider hidden nutritional deficiencies as BMI is a poor surrogate marker for malnutrition and many individuals with normal or even high BMIs have micronutrient deficiencies, which also play a role in increasing risk of TB [21].

Further work is also warranted on the performance of various TB testing modalities in the context of malnutrition. Much like HIV/AIDS, N-AIDS also blunts the immune response and alters the inflammatory milieu, which can impair the performance of tests for screening or diagnosis of TB [3, 5]. This is immediately evident in testing for latent tuberculosis, where the tuberculin skin test produces a smaller induration in severely malnourished individuals, particularly in children [22]. Malnutrition also appears to lower the sensitivity of interferon gamma release assays in children [23]. The impact of malnutrition on the sensitivity and specificity of potential triage tests such as C-reactive protein, as well as established tests like nucleic acid tests, may also need to be studied in malnourished populations [24]. Without optimizing our testing for malnourished populations, we are likely to continue missing TB disease in millions.

The WHO has recommended bidirectional screening for HIV and TB [2]. Given that TB prevalence is higher among malnourished individuals and a large proportion of people living with TB are malnourished, there is a critical potential role for concurrent screening for malnutrition and TB. Unfortunately, BMI, which is often used as a measure of malnutrition, is not an ideal indicator, particularly in the context of South and Southeast Asia [25]. Nutritional assessment for PWTB with anthropometry, even though metrics like BMI are imperfect nutritional measures, should be the minimum standard given the prognostic value of nutritional status [14]. In high-burden settings, there may even be a BMI cutoff for certain anthropometric indicators such as weight loss above which the prevalence of TB is high enough to justify periodic screening. This screening could involve lower sensitivity tests such as radiography or even nucleic acid amplification tests, which could be pooled from a household level. Additional epidemiological studies and health economic models can help guide decision-making regarding the model, frequency, and nutritional metrics to guide cost-effective TB screening in populations with a high burden of malnutrition. Acknowledging that malnourished individuals are a key and vulnerable population for TB will help hone our detection efforts.

### Treatment of TB

Research, from discovery to implementation, in TB malnutrition has been mired in a form of collective ennui. This is in large part due to the perception that correcting malnutrition, a socially driven and multifactorial entity, is too structural to undertake. “How does one prevent malnutrition related TB without tackling poverty?” one may naturally wonder. Yet, we must recall our recent past when weight gain through calorie-dense foods was *the* cornerstone of TB therapy in sanatoria [6]. After the landmark Madras study showed that outpatient pharmacotherapy was comparable to care in sanatoria, TB elimination efforts focused almost entirely on detection and treatment efforts [26]. Although success with combination drug therapy had been demonstrated, those who were malnourished continued to suffer worse health outcomes than others, even in the wake of such treatment.

A large multicenter prospective cohort analysis found that severely malnourished individuals are at twofold higher risk of unfavorable treatment outcomes including death, treatment failure, relapse, and treatment discontinuation: adjusted incidence rate ratio (aIRR) = 2.05; 95% CI 1.42–2.98 [14]. Lack of weight gain during TB therapy was also associated with increased risk of these outcomes (aIRR = 1.81; 95% CI 1.27–2.61). Severe malnutrition was associated with a fourfold increased risk in mortality (aIRR = 4.17; 95% CI 1.97–9.52), and lack of weight gain during therapy was associated with a fivefold increased mortality risk (aIRR = 5.18; 95% CI 1.52–17.69). These findings corroborate those of numerous prior studies which have been previously reviewed [14].

Malnutrition has been identified as a risk factor for tuberculosis incidence, severity, and treatment failure. There are numerous reasons for the worse outcomes seen among malnourished individuals. There is direct evidence from studies in animals and humans that malnutrition impairs the immune response to TB [3, 5]. This may manifest in more severe disease. Indeed, studies have linked malnutrition to increased lung involvement and cavitation [27]. Of course, given the bidirectional relationship of TB and malnutrition, it is difficult to ascertain the causality. Malnutrition may also affect pharmacotherapy. Malnourished individuals, particularly children, may have subtherapeutic pharmacokinetic exposure to key drugs such as rifampin and isoniazid [28, 29]. Importantly, malnourished persons are also at increased risk of supratherapeutic concentrations and associated toxicity from drugs like ethambutol and aminoglycosides [30]. Heartbreakingly, some PWTB discontinue their TB therapy because their appetites reappear during TB therapy and they lack access to food [31]. This is illustrated in the following quotation from a mixed methods study in Lesotho:

“The challenges that I have recognized is that they make you feel very hungry more often. Sometimes that is bad because there are no piece jobs [daily work] at all. You find that you eat a lot in a short space of time, that doesn’t sit well with the wife even though she won’t say it, but you will recognize her displeasure” [31].

Given the prognostic value of nutritional status, serial assessment of nutritional status should be a standard component of TB therapy, akin to HIV testing. This can be done inexpensively using anthropometric indices such as weight monitoring and mid-upper-arm circumference. The neglect of malnutrition in TB management is evident in that we are still using simple anthropometric measures for N-AIDS while we can readily measure T-lymphocyte subsets and viral loads for persons with HIV and TB co-infection. We have prioritized neither implementation or access to tools for measuring an individual’s nutritional status, nor tailored nutritional interventions as part of TB treatment or screening programs.

Further, malnourished individuals living with TB should receive nutritional screening and counseling, as well as nutritional supplementation with macronutrients and micronutrients [32]. Although a Cochrane review of randomized trials of nutritional interventions for PWTB did not find evidence of improvement in cure or mortality, quite importantly, the meta-analysis was insufficiently powered [33]. Two non-randomized interventional studies of nutritional interventions in India, with considerably more participants than in all the studies pooled in the Cochrane review, demonstrated significant improvements in treatment success [34, 35].

To a large extent, the higher treatment success rate among PWTB is attributable to reductions in disengagement from care. Indeed, in a study among PWTB in West Bengal who were below the poverty line, only 1/173 (<1%) individuals receiving nutritional support were lost to follow up as compared to 41/400 (10%) participants in the control group [36]. Nutritional support may support adherence and preserve effective linkages to care. The reasons for improved retention in care may be both biologic and economic, as nutritional support can ease financial strain for households with TB and reduce food insecurity [37–39]. Thus, nutritional support can help attain the End TB strategy’s goal of eliminating catastrophic costs associated with TB.

We contend that discussions on the role of nutritional support for PWTB often fixate on the anticipated improvement in TB treatment outcomes. Doing so detracts from the idea that malnutrition is a widely prevalent comorbidity that affects the functional and economic recovery of persons with TB. Independent of its effects on TB disease outcomes, malnutrition deprives people of their full potential as acknowledged by the Copenhagen Consensus. Malnourished individuals face higher direct costs through increased health care needs and impaired cognitive function in children and decreased physical productivity in adults [40].

Malnutrition remains a critical comorbidity with tangible impacts on TB treatment. Pillar 1 of the End TB strategy seeks to provide integrated, person-centered care [41]. Part of this is providing collaborative management of comorbidities. Few would dispute that malnutrition is a common comorbidity among PWTB. Therefore, nutritional assessment and support should be integrated into standard TB therapy. Not doing so is a failure of care.

### Prevention of TB

We continue to seek the missing millions with TB because of the biomedical approach’s failure to address upstream factors. Decades of data link nutritional status of populations to TB incidence and mortality.

During the two world wars, numerous natural experiments highlighted the connections between malnutrition and TB [5]. During the Dutch famine (1944–1945), when the daily caloric intake for the average adult approximated a mere 600 kcal/day, there was also a dramatic increase in TB mortality [42]. Conversely, the German blockade of Denmark during World War I, which prevented the Danish from exporting meat, fish, and dairy products, led to a surplus of these food items. In the following months, TB rates fell drastically in Denmark even as the incidence climbed in nearby warring countries [5].

Prospective cohort analyses have supported evidence from ecological studies during the world wars. An analysis of the First National Health and Nutrition Examination Survey (NHANES I) reported a twelvefold increase in incident TB among underweight Americans (BMI <18.5kg/m^2^) [43]. This estimate is not far from the WHO’s estimate that HIV increases the risk of TB approximately twentyfold [2]. A systematic review of prospective cohort studies proposed a log-linear relationship between BMI and TB incidence [44]. Between the BMI range of 18–30 kg/m^2^, each 1 kg/m^2^ increase in BMI was associated with a 13.4% lower risk of incident TB. It is possible that the relationship between BMI and incident TB becomes nonlinear at lower BMIs [45]. These findings are supported by more recent prospective cohort studies of household contacts of PWTB that found that underweight contacts had increased transcriptomic TB risk signatures, indicating a higher risk of progression to active TB disease, compared to their normal weight counterparts [46].

While the literature on malnutrition and TB has largely focused on studies using BMI as a metric for malnutrition, some compelling data also link micronutrients to TB. A prospective cohort from Peru showed that individuals with baseline vitamin A deficiency (serum retinol <0.70µmol/L) had a tenfold higher risk of TB disease: adjusted odds ratio (aOR) = 10.53; 95% CI 3.73–29.70, and those deficient in vitamin D (serum α-tocopherol <5.00 mg/L) had double the risk (aOR = 2.29; 95% CI 1.29–4.09) [47, 48]. As reviewed previously, micronutrient supplementation alone has not been demonstrated to reduce TB incidence [33]. While some studies were underpowered, even large randomized studies, such as one of vitamin D supplementation that included 6250 individuals, did not find evidence of reduction in incident TB: hazard ratio (HR) = 0.78; 95% CI 0.54–1.13, or TB mortality (HR = 1.04; 95% CI 0.85–1.25) [49]. This is not altogether very surprising however, as severely malnourished individuals often have numerous nutrient deficits. Macronutrients and micronutrients play distinctive roles in the adaptive and innate immune response [50]. Rescuing just one nutrient through a vertical public health program may be easily implementable, but will likely not be sufficient to control N-AIDS.

The role of food as medicine or prevention should not be dismissed out of hand. Modeling studies have demonstrated that even modest reductions in malnutrition on a population scale through government action could lead to meaningful reductions in TB incidence and mortality, particularly in South Asia and Southeast Asia [51, 52]. However, conversations on population-level nutritional interventions often stagnate due to concerns surrounding costs and sustainability. This represents a failure of imagination. Indeed, similar skepticism existed in the 1990s around the feasibility and sustainability of antiretroviral therapy in low- and middle-income countries [53, 54]. A combination of advocacy, innovation, and global health investment realized the possibility of early antiretroviral therapy in low-income countries, transforming the lives of millions [54].

More recently, the Reducing Activation of Tuberculosis by Improvement of Nutrition Status (RATIONS) study provided evidence that nutritional support can reduce TB incidence and transmission [55]. The study randomized more than 10,000 household contacts of PWTB to either receiving nutritional support in the form of a food basket that provided 750 kcal per day (including 23g of protein) and micronutrients or the control group. In both the intervention arm and the control arm, PWTB received 1,200 kcal of food per day. The investigators found a 40% reduction in incident TB over 2 years of follow up: adjusted incidence rate ratio (aIRR) of 0.61(95% CI 0.43, 0.85). To be sure, the RATIONS study was conducted in a region of India with immense socioeconomic deprivation and a low incidence of HIV. As such, the observed effect size may not apply to other contexts. With that said, these dramatic results demonstrate that food is an important adjunct to TB vaccines that we already have, and TB due to malnutrition is preventable.

A health-economic model that evaluated a hypothetical population-scale reduction in malnutrition through India’s national subsidized ration program further reinforced the importance of focusing upstream. The model projected that such an intervention could precipitate large reductions in TB incidence and mortality over a 5-year time horizon while being highly cost-effective. The incremental cost-effectiveness ratio (ICER) was $470 per disability adjusted life years (DALY) averted [56]. The intervention was more cost-effective among persons with HIV and household contacts of PWTB compared to the general population. Importantly, the cost-effectiveness in the model was driven in large part by reductions in the DALYs associated with malnutrition. Although population-scale nutritional interventions involve large budgets, they also have numerous positive externalities beyond simply reducing TB.

Given the complexities and the knowledge gaps involved in the procurement, delivery, and utilization of food, some may argue that we should simply hold out for a more traditional TB vaccine. But there is no reason to consider nutrition and vaccine as mutually exclusive approaches. After all, vaccines are more likely to be effective in well-nourished individuals [57]. The immunology of malnutrition, both macronutrients and micronutrients, must be better characterized to ensure that TB vaccines are equally efficacious among malnourished persons [57]. Further clinical trials should assess if higher doses of vaccines are needed among malnourished individuals or if nutritional supplementation with multiple micronutrients could increase vaccine efficacy. Given the large prevalence of malnutrition in high TB-burden countries, ensuring that any new vaccine candidate is effective in this key and vulnerable population is critical.

### Moving things forward

It is time to recognize that malnutrition is a critical TB comorbidity. Given the prognostic value of BMI in PWTB and the ease and inexpensiveness of conducting anthropometry, TB programs must integrate nutritional screening for TB at diagnosis and during treatment just as they routinely screen for HIV infection in all PWTB. In addition to macronutrient deficiency, programs should also standardize testing for anemia, which is common among PWTB and linked to worse outcomes [58]. Importantly, malnutrition is not the same as food insecurity. Malnourished PWTB should also be assessed for reversible causes of malnutrition such as digestive disease, enteropathogenic infection, substance use disorders, chronic diseases, and psychiatric disorders.

Nutritional screening is already recommended by the existing WHO guidelines for nutritional support of PWTB, but has not been implemented widely [32]. At minimum, we believe that BMI at diagnosis and weight change after intensive phase of therapy should be systematically reported in the WHO’s annual global TB report alongside statistics on HIV-TB. Recording and reporting rates of malnutrition among PWTB can shed greater light on the magnitude of the problem and prompt greater action on TB undernutrition just as it did for TB-HIV [59].

The International Union Against Tuberculosis and Lung Disease’s TB undernutrition working group recommends that nutritional interventions should provide balanced and diverse diet rather than an isolated nutrient [60]. Providing nutritional support can improve food access, but some PWTB may still struggle with utilizing the provided supplements optimally. Therefore, providing nutritional counseling alongside in-kind or cash support can enhance nutritional outcomes. Nutritional counseling is recommended by the WHO guidelines, but is not routinely offered to PWTB [32]. At the end of TB therapy, programs should make efforts to help patients utilize the social welfare benefits to which they are entitled such as ration programs.

While several national TB program managers want to provide nutritional support to PWTB, they lack funding, as well as the logistical and technical support to do so. TB programs should request and prioritize funding from the global fund and other funders for nutritional counseling and support for PWTB and their household contacts, particularly those with severe malnutrition. Countries like Benin already do this and more should follow their lead. Existing infrastructure could be adapted and scaled up for robust action on malnutrition among PWTB and their household contacts as well as other key and vulnerable populations. For instance, India already has an infrastructure for delivering cash transfers, in-kind rations, mid-day meals, and nutritional counseling [61–64].

Given historical data, model projections, and now empiric evidence from the RATIONS study, it is clear that we can cost-effectively make major gains in global TB elimination efforts by reducing the prevalence of malnutrition in the populations of high TB burden countries [5, 52, 55, 56]. However, addressing population-level malnutrition extends beyond the scope of national TB programs. It necessitates a coordinated response across various sectors including government agencies and ministries focused on health, food, education, finance, energy, and agriculture. Additionally, involving academic and private sectors can help improve the design and reach of interventions. Some examples of such concerted action by governments can be seen in Ethiopia, Nepal, Brazil, Thailand, Peru, Vietnam, Bangladesh, and Senegal [65–71]. While the focus of national nutritional programs is often on children and pregnant women, malnutrition across the lifespan should be addressed to reduce TB incidence.

On an international scale, the WHO and national TB programs are ill-equipped and ill-funded to address malnutrition as a key comorbidity and determinant of the TB pandemic alone. In part, this is why the global TB elimination efforts have been largely centered on biomedical interventions. The problem is too large to address in siloes. Closer collaboration between bodies such as the WHO, USAID, UNICEF, Global Fund, World Food Programme, World Bank, and International Monetary Fund is crucial to propel the TB malnutrition agenda. This would not be a novel concept as the World Food Program has a history of supporting Afghan households with TB with in-kind supplementation [72]. Facilitating cross-organizational support is critical and urgent. The WHO Multisectoral Accountability Framework to accelerate progress to End TB (MAF-TB) may serve as a valuable tool in identifying and delineating the roles of stakeholders in this coordinated effort [73].

Further, malnutrition is a cross-cutting issue with implications for numerous global health and development priorities and is indeed relevant to one of the sustainable development goals (SDG): zero hunger (SDG2) [74]. TB advocates should join hands with advocates for HIV, respiratory diseases, maternal and child health, neglected tropical diseases, women’s empowerment groups, and others to present a united front. Because funding for such efforts may be a point of contention, joint advocacy highlighting such possibilities is imperative and more likely to be successful [75].

Researchers can do their part by conducting screening and collecting data on nutritional status as part of prospective studies in the same manner they would for HIV status. Given the depth of evidence regarding the importance of nutritional status for TB incidence and clinical outcomes, not doing so creates the risk of unmeasured confounding. Further, more studies are needed to understand the nutritional needs of adults, children, and pregnant people with TB, with the caveat that these may vary based on culture and context. Increased funding allocations for research devoted to nutrition and TB is imperative, particularly for operational research that allows us to optimize nutritional interventions and delivery mechanisms.

## Conclusions

In 1915, the New York City’s Department of Public Health asserted that “the city can have as much reduction of preventable disease as it wishes to pay for. Public health is purchasable; within natural limits a city can determine its own death rate” [76]. A century of data has consistently pointed to malnutrition as a key driver of the TB pandemic. However, unlike HIV, assessment and care for malnutrition has not been prioritized among PWTB and those at risk for TB. To ensure the success of the End-TB strategy, we must acknowledge malnutrition as a dominant and pervasive driver of TB disease and poor TB treatment outcomes. Fortunately, malnutrition is a modifiable factor with simple, not simplistic, interventions. Meaningful reductions in TB incidence and mortality are achievable through investments in nutrition. The global health community has previously tackled one form of AIDS through inter-disciplinary action. We can do the same for N-AIDS as we detect, treat, and prevent TB.

## Data Availability

Not applicable.

## Abbreviations

AIDS: Acquired immunodeficiency syndrome
aIRR: Adjusted incidence rate ratio
aOR: Adjusted odds ratio
BMI: Body mass index
DALY: Disability-adjusted life years
HIV: Human immunodeficiency virus
ICER: Incremental cost-effectiveness ratio
N-AIDS: Nutritionally acquired immunodeficiency syndrome
NHANES-1: First National Health and Nutrition Examination Survey
PAF: Population attributable fraction
PWTB: Persons with tuberculosis
TB: Tuberculosis
USA: United States of America
WHO: World Health Organization
